# On the Medical Use of Alkalis

**Published:** 1801-06

**Authors:** 


### *On the Medical Use of Alkalis, by* Dr. Stutz.

[ Continued from our laft, pp. 219—223. ]

On the 13th of July, 1800, I was fent for to vifit a young woman, 23 years of age. I found her in bed, feized with the moft violent convulfions. The extremities were bent and turned in feveral directions, and when the convulfions remitted in the extremities, the trunk, and particularly the breaft, was attacked with great violence, and a fhort, frequent, and laborious breathing, attended with fingultus, came on; the jaws were locked, and the otherwife frefh and blooming countenance of the woman grew pale and cold at each paroxyfm. Both hands and feet were likewife cold, and of a bluifh colour. Pulfe fmall, weak, and irregular. Befides thefe fymptoms, the patient became deprived of fpeech; and though fhe remained during the paroxyfm perfectly fenfible, yet fhe could not utter one fyllable. Under thefe circumftances I could not acquire any information refpecting the caufe of the prefent difeafe, particularly, as the parents affured me fhe had always been quite well till this day, when, on coming from church, fhe had fallen down in a fwoon, after which the convulfions enfued. I had therefore no other indication than that of employing fuch remedies as might reftore the irregular action of the organic fibre to an equilibrium, or, in other words, which act as antifpafmodics; for this purpofe I thought I could not choofe a better remedy than the alkalis, after what I had experienced from them in one of the moft violent fpafmodic difeafes, the tetanus. I prefcribed, therefore, one drachm of purified vegetable alkali, (fal tartari) diffolved in four ounces of water, of which I ordered a table fpoonful to be taken every quarter of an hour. As I purpofed to try the alkalis by themfelves, I gave no opium nor any other remedy. It was impoffible to prevail on the parents to put her into a hot bath impregnated with kali. About eight o'clock in the evening I was called again, when I found that though the convulſive paroxyſms had abated for ſome time after the medicine, they had recurred about an hour ſince with increaſed vehemence. The patient ſuffered the moſt violent diſtortions in the extremities; and when theſe remitted, the reſpiration became convulſive and laborious to ſuch a degree that the body was moſt violently thrown up. As theſe ſymptoms were leſſening, the patient breathed with great difficulty and much rattling, the countenance grew pale, the whole body became cold, the pulſe weak, and every body who ſaw her, imagined her diſſolution to be very near. But on a ſudden, the convulſive fits wreſted and diſtorted both hands and feet; and it was indeed aſtoniſhing how thoſe violent actions could be excited in a body that was almoſt exhauſted. In this way the fits continued in an uninterrupted ſeries; and an apparent ſtruggle between life and death, kept her friends in continual ſuſpenſe. In this deplorable ſituation, however, I became not diſpirited, but being reſolved to leave nothing untried that could reſuſcitate the dying flame of life, I endeavoured to give her ſome table ſpoons full of the alkaline ſolution, which ſhe had not taken for above one hour, the parents expecting her death to be inevitable; after which I ordered warm fomentations of ſoap water with lapis cauſticus to be applied on the belly, and the whole body to be waſhed and rubbed with the ſame ſolution; and two table ſpoons full of the above alkaline ſolution to be continued every quarter of an hour. Before I left her, ſhe had another violent fit, that was probably excited by holding ſtrong ſpirits of hartſhorn under her noſe. The vehemence of this paroxyſm abated however by degrees, and the patient lay with a ſhort, hurried, and rattling reſpiration.

On the morning of the 14th, I was told that ſhe had frequently enjoyed quiet intervals, though the fits had as often recurred, with more or leſs violence. The fomentations had been diligently applied, not ſo the waſhing and frictions. She had taken a little broth that morning. When I ſaw her, ſhe had ſhortly before been ſeized by another vehement fit, which being over, ſhe found herſelf in a weak, and, except ſome difficulty in breathing, in a quiet ſtate, and was quite ſenſible of what paſſed around her. I aſked her very cautiouſly about the cauſe of her preſent diſeaſe, and heard, that after dinner ſhe had over-heated herſelf with running very faſt on a hot day, after which her noſe began to bleed, and in order to ſtop the hæmorrhage, ſhe had drawn cold water into the noſe, and waſhed her head and neck. Some time after ſhe had fallen into a ſwoon, out of which ſhe was rouſed by the preſent

ſymptoms.

ſymptoms. As ſhe was thus lamenting her ſituation, ſhe was ſeized by a very violent convulſive fit. I gave her immediately a doſe of the alkaline ſolution, when the convulſions ſoon abated, though the hands remained ſtiff and ſpaſmodically diſtorted. I preſcribed now two drachms of the kali to be diſſolved in ſix ounces of water, of which I ordered alternately, one or two table ſpoons full to be given every half hour, together with the continuation of the warm alkaline fomentations. About the evening of the ſame day I viſited her again; but how much was I aſtoniſhed, when I ſaw the room crouded with people, kneeling and praying, and the clergyman attentively engaged to prepare her for death, an event that was almoſt inſtantly expected. This ſcene however, ſurpriſed me exceedingly, as I had reaſon to hope from the ſituation I left her in the ſame morning, that ſhe was far advanced towards her recovery. I was then informed, that the patient had, a few quiet intervals excepted, been attacked by very laſting fits of convulſions, whereby ſhe had grown ſo weak, that they imagined ſhe would die every moment. On feeling her pulſe, I found it as weak and quick as yeſterday, and ſometimes intermitting. Though I conſidered her not to be in ſo bad a condition as the people about her fancied, I thought myſelf induced to quit the plan of curing this convulſive diſorder with alkalis only, and proceeded to the alternate application of alkali and opium. I preſcribed therefore *two* drachms of laudan. liquid. Sydenham, of which from ten to ſixteen drops were alternately given with the alkaline ſolution, and beſides this an emollient clyſter impregnated with alkali was applied.

The 15th, ſhe was much better, as the convulſive fits had almoſt entirely abated; after having three times given the doſe of the drops, the pulſe was ſtronger, undulated, and the countenance more lively. In lieu of the convulſions a ſpaſmodic trembling of the extremities had ſupervened, and ſhe complained at the ſame time of a dullneſs and inſenſibility in the limbs. A warm perſpiration ſurrounded the body, and it was not till after ſhe had ſuffered more than the anguiſh of death, that the patient began to recover a little. She took ſome broth and coffee with much appetite. I now began to doubt whether the opium by itſelf, without the aſſiſtance of the alkali, had not effected this remiſſion of the diſeaſe, and conſequently, in order to put the matter to a fair trial, I ſuffered no alkaline ſolution to be taken that day, but only to continue with the laudanum, twelve drops four times a day; the diet conſiſted of broth with the yolk of an egg, ſome coffee, and the emulſion of ſweet almonds. On viſiting the patient the ſame evening, I found her in a ſtrong perſpiration with an undulating pulſe, and though the convulſions had not recurred, yet the trembling of the limbs was conſiderably increaſed, the reſpiration more difficult; ſhe complained of a painful ſhortneſs of breath, being at the ſame time very anxious and fearful that the convulſions might return. Under theſe circumſtances, I thought it not proper to diſcontinue the alkali any longer, and I preſcribed therefore a drachm and a half of pure potaſh in ſix ounces of water, of which every hour a table ſpoonful was to be taken, and of the laudanum eight drops only every two hours. A warm pediluſe of common lye was likewiſe ordered.

The 16th in the evening, the patient found herſelf pretty well, as ſhe had ſlept quietly for ſeveral hours, and only been once attacked by the ſpaſmodic trembling. The pulſe ſtrong and undulated. I ordered her to take two table ſpoons full of the alkaline ſolution three times a day, and eight drops of the laudanum twice; an emollient clyſter, containing ſome alkali was applied, her bowels having not been moved for above three days. In the evening ſhe was pretty well, and had ſuffered only two fits of the ſpaſmodic trembling.

The 17th, 18th, and the following days the patient grew by degrees better, the trembling ceaſed, and a dull ſenſation in the limbs only remained, a natural conſequence of the immoderate efforts of the muſcular and nervous ſyſtem, for which I recommended a ſtrengthening diet, and ſix ounces of aqua cinnamomi c. vino with one drachm of ſal tartari, and half a drachm of laudanum, of which ſhe was to take two table ſpoons full three times a day, and ſhe was allowed to drink wine mixed with a little water. In this way ſhe daily got better, and about eight or ten days after the firſt attack of the convulſive fits, ſhe walked about again, to the utmoſt aſtoniſhment of every body who had ſeen her in the convulſions.

4. I have ſeen two or three inſtances of *Cardialgia* in delicate women, ſubject to ſpaſmodic ſymptoms, where a few grains of ſal tartari diſſolved in camomile or cinnamon water, brought almoſt inſtant relief in removing the painful paroxyſm. Care ought however to be taken, not to give in the beginning too large a doſe of the alkali, for fear of cauſing an over irritation of the ſtomach, and other diſagreeable ſymptoms. But ſhould no relief be produced, after having conſumed one drachm, or one drachm and a half, we ought to proceed to the alternate application of alkali and opium. I have had a caſe of a pregnant woman very much inclined to cardialgia, who was troubled during the whole time of her pregnancy with heart-burn, vomiting, &c. for which complaints, mineral waters, fixed air, magneſia, lapides cancrorum, ginger, &c. had been employed without much avail, as the the complaint always recurred a ſhort time after. I preſcribed therefore the ſal tartari diſſolved in aq. cinnamomi ſine vino, upon which the complaints left her entirely for two days, but after this time they recurred. I increaſed the quantity of the alkali to one drachm and a half, by which the patient got relieved again; and in this way the proportion of the alkali was brought to two drachms and a half. This doſe however cauſing a burning in the mouth and throat, and being at the ſame time of leſs effect, I diminiſhed it to *two* ſcruples per diem, to be taken at four different times, and twice in the intervals ſeventy drops of laudanum, both which remedies cured, in one day, the heart-burn, vomiting, &c. for a conſiderable time, till they were again rouſed by an accidental cauſe; but they ceaſed upon employing the ſame remedies. This caſe ſhowed the moſt ſtriking analogy with the experiments of Mr. Humboldt, on the influence of chemical ſubſtances on excitability. The cardialgia was in the beginning ſolely removed by alkalis; but being again excited by any adventitious cauſe, the alkali, even in an increaſed doſe, proved of no avail, but opium inſtantly relieved her. When, however, I intended to remove the pain by opium merely, it anſwered the purpoſe in the beginning, but when continued afterwards, it had no effect, even in larger doſes. Alkali however, now given, operated to my expectations. This experiment I have purpoſely repeated three times, and the reſults were always the ſame; the alternate application of opium and alkali had always the expected effect; a fact that is likely to prove moſt advantageous to the medical art, in curing the moſt dangerous complaints.

5. I have had a caſe of ſpaſmodic aſthma, which the vegetable alkali ſolely removed. Different remedies, and the moſt efficacious antiſpaſmodics had been tried in vain, till, when quite at a loſs what to do, I had recourſe to the oleum tartari per deliquium, which I gave by drops in increaſed doſes; but I ſhall communicate my remarks on this caſe at another time.

Although I have hitherto had no opportunity of making experiments with alkalis in the following *general* ſpaſmodic diſeaſes, yet I am led to conclude from analogy and induction, and upon ſome other good grounds, that great uſe may ariſe from employing alternately the opium and alkali, 1. In *hydrophobia*, alſo ſoon after the bite, in order to prevent the breaking out of the diſeaſe; 2. In *epilepſy*; 3. In *catalepſy*; 4. In *the St. Vitus's dance*, (*chorea St. Viti*) in all which diſeaſes I poſitively believe that the alkali, alternately adminiſtered with opium, and externally applied in a warm bath, will prove moſt efficacious, particularly in hydrophobia, a diſeaſe which ſeems ſo much to agree in ſeveral points with tetanus; and I am inclined to think, that the rational and timely application of this method of cure is likely to be crowned with succefs, in a difeafe that generally fruftrates medical affiftance.

Having communicated my ideas and obfervations on the great ufe of alkalis in thofe general difeafes, I proceed now to ftate their efficacy in feveral topical complaints, when externally applied. 1. *In palsies* occurring after previous apoplexies, of which I have cured two cafes with alkali: The paralytic limb, arm, or foot, was put into a warm bath of common lye, with the addition of fome lapis cafticus; the quantity was increafed by degrees, and after thefe repeated topical baths, the paralytic part received again its fenfation and power of moving. In afthenic gouty complaints, in the ifchiadic paffion, thofe warm baths may moft probably prove of very great fervice, and likewife in any weaknefs that frequently remains after fits of gout. 2. The cauftic alkali is alfo of avail in foul afthenic ulcers, of the foft as well as of the folid parts, on which it is applied after being diffolved in a fufficient quantity of diftilled water: The affected part is, for feveral minutes a day, put into a warm folution of lapis caufticus chirurgorum, of which, in the beginning, *one* or *two* grains are put to *one* ounce of water. At firft it caufes fome pain, which, however, by continued application ceafe, even when the quantity of the lapis caufticus is increafed: the ulcers become by that means clean, and the cure is uncommonly forwarded; they are afterwards only dreffed dry, or with lint dipped into a diluted folution of lapis caufticus. Of the fuccefs of this cure I have had frequent experience, and I fhall not omit communicating in future, the cafes that fall under my obfervation. Many will doubt the good effect of alkalis in caries, particularly thofe who are unacquainted with the experiments of the celebrated *Dr. Lentin*, of Hanover, with the phofphoric acid, which this gentleman found of much fervice in that affection; but I truft they will only retain their doubts till I fhall have laid before them my ideas on the mode in which the alkalis feem to act upon organic bodies. I have endeavoured to draw the attention of medical men to the great ufe of alkalis in feveral dangerous difeafes, by relating to them what I have experienced in my own practice, and I fhall at a future time take an opportunity of communicating my ideas on their mode of action. I am confcious, that what I have ftated here is not entirely new; alkalis were formerly much employed, though the theory which indicated their ufe was far different. The phyficians of the time of *Helmont* and *Sylvius* employed them with the idea of fubduing the exceffive acidity of the humours that threatened to deftroy the conftitution; others ufed abforbents, ents and alkalis, to refiſt the ſeptic tendency of the blood; and but lately, *Dr. Barker** recommended lime water and ſal abſinthii againſt febrile diſeaſes, deriving, according to the ingenious theory of *Dr. Mitchill*, the material cauſe of fevers from the oxydated azotic gas, or, as he calls it, ſeptic acid.

Many paths lead to truth, but to go the ſtraighteſt and ſhorteſt way is granted to few mortals.

* See Medical and Phyſical Journal, Vol. I. p. 274.

